# Construction Notes

**Published:** 1896-02-01

**Authors:** 


### CONSTRUCTION NOTES.

### New Ward at the Corbett Hospital.

Plans for the new ward at the Corbett Hospital, Brierley Hill, show that though this has popularly been called "The Children's Ward," this is a misnomer, for although the provision of extended accommodation in the hospital is intended to provide a new ward specially for the treatment of children, the new ward will not be devoted to that purpose. Sixty male patients, who now occupy the male medical ward, will be transferred into the new building, which adjoins the accident ward, so that by this arrangement the two wards for male patients will be on the same side of the hospital; the children being accommodated in what is now used as the men's medical ward. The new building is at the northern end of the hospital. It is one storey in height, and its dimensions are 32 ft. by 20 ft. It is accessible by an enclosed archway, with a glazed door, from the accident ward. Accommodation will be provided for eight patients. The hot-water apparatus has been extended to the additional room, and a sink and lavatory are provided. Several children's cots are being provided in the women's ward of the hospital, and these will be removed to the ward now to be devoted to children. The cost of the building will be upwards of £400. Mr. Thomas Robinson, of Stourbridge, is the architect, and Mr. W. North builder and contractor.

